# A Case of Hereditary Leiomyomatosis and Renal Cell Carcinoma

**DOI:** 10.1155/2016/3793986

**Published:** 2016-04-07

**Authors:** Sarah Mehrtens, David Veitch, Elizabeth Kulakov, Conal M. Perrett

**Affiliations:** University College London Hospital, 235 Euston Road, London NW1 2BU, UK

## Abstract

A 49-year-old lady presented with multiple recurring painful lesions over her thighs, arms, and back. Past medical history included a left sided nephrectomy for renal cell carcinoma and a hysterectomy for multiple uterine fibroids (leiomyomas). Histopathological examination revealed changes consistent with pilar leiomyomas. Gene mutation analysis confirmed a diagnosis of hereditary leiomyomatosis and renal cell carcinoma. Hereditary leiomyomatosis and renal cell carcinoma is an uncommon autosomal dominant condition characterised by the concurrent presentation of cutaneous and uterine leiomyomas. Renal cell carcinoma associated with this condition is more aggressive and a significant cause of mortality. Due to this association with potentially fatal renal cell carcinoma we felt that it was important to highlight this case with an update on pathophysiology and management.

## 1. Introduction

We present an interesting case of a woman who presented to our dermatology department with specific cutaneous findings. Following further important information elicited in the history, detailing associated systemic involvement, we confirmed our diagnosis of hereditary leiomyomatosis and renal cell carcinoma (HLRCC), an uncommon genodermatosis, with genetic testing. Due to the association with potentially fatal renal cell carcinoma we felt that it was important to highlight this case as a reminder to other dermatologists.

## 2. Case Presentation

A 49-year-old woman presented with multiple recurring lesions over her thighs, arms, and back. These lesions had developed over several years and were tender to touch. Several of these lesions had been excised and there was evidence of recurrence at several previous excision sites.

Past medical history included a hysterectomy for multiple uterine fibroids and a left sided nephrectomy for renal cell carcinoma.

**Figure 1:**
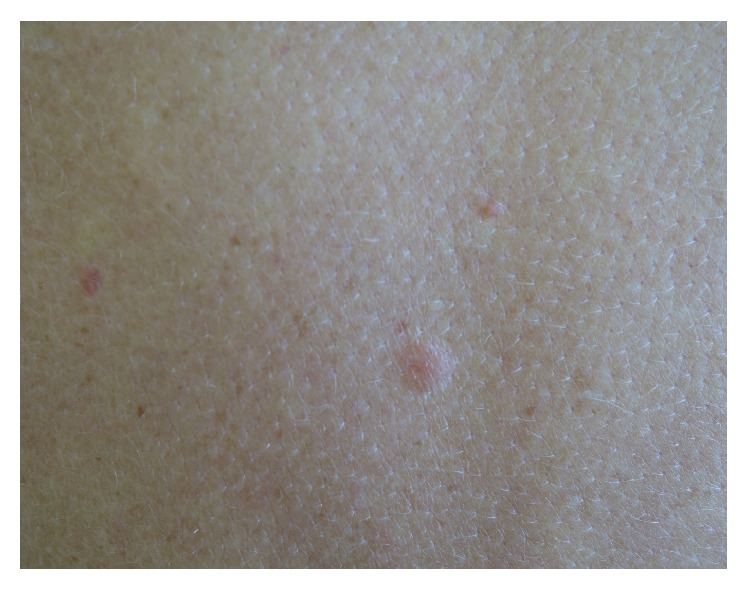
Multiple firm tender reddish nodules on the arms.

Examination revealed multiple, firm, reddish nodules on the upper back, arms, and thighs (Figure 1).

## 3. Investigations

**Figure 2:**
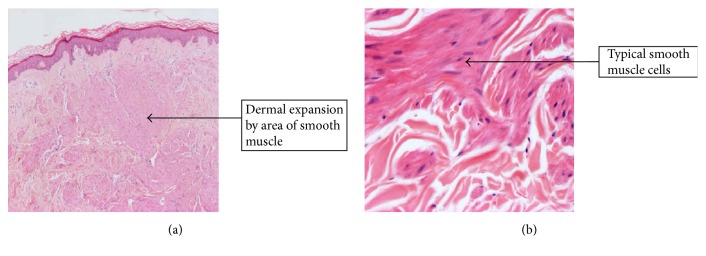
(a) H&E stained section shows an ill-defined proliferation of smooth muscle in the upper to mid dermis; (b) higher power of typical smooth muscle cells of the lesion without atypia or pleomorphism; (a) ×4; (b) ×20.

Histopathological examination revealed ill-defined proliferation of typical smooth muscle cells in the upper to mid dermis, consistent with pilar leiomyomas (Figure 2).

Abdominal magnetic resonance image (MRI) showed evidence of a previous left nephrectomy, angiomyolipomas in the right kidney, and multiple tiny simple cysts in the liver.

Gene mutation analysis revealed that the patient was heterozygous for a pathogenic mutation in exon 3 of the fumarate hydratase (FH) gene.

A diagnosis of HLRCC was made.

## 4. Discussion

HLRCC is an uncommon autosomal dominant condition characterised by the variable presentation of multiple cutaneous leiomyomas, uterine leiomyomas, and renal cell carcinoma [1]. It is also known as MCUL (multiple cutaneous and uterine leiomyomatosis) and Reed's Syndrome, following first documentation of the condition by Reed in 1973. Nearly all affected women present with uterine fibroids and approximately 75% present with cutaneous manifestations [2–4]. Several reports, however, have stated that approximately 40% of HLRCC patients have only mild cutaneous features (<5 leiomyomas) or lacked skin lesions [5–7]. The penetrance of renal cancer in HLRCC patients is incomplete and is generally reported as being low. However, multiple studies have shown that it is exceptionally aggressive and a significant cause of mortality [8–13].

The exact pathogenesis is unclear but it is now well established that HLRCC is caused by a heterozygous mutation in the gene encoding for fumarate hydratase (FH) on chromosome 1q43 [3, 4, 8]. FH is an enzyme in the tricarboxylic acid cycle that converts fumarate to malate and is thought to act as a tumour suppressor gene. In HLRCC-associated tumours, the somatic inactivation of the remaining FH allele causes functional loss of FH leading to abnormal intracellular accumulation of fumarate, resulting in tumourigenesis. The majority of mutations are missense mutations, but studies have also identified nonsense mutations, frame shifts, and whole gene mutations [14].

Presentation of HLRCC is heterogeneous due to poorly understood epigenetic effects. Leiomyomas start developing from adolescence to middle age with a mean age of presentation of 25 years and typically affect the trunk, extremities, and face. These benign tumours arise from the arrector pili muscle, genital muscularis tunica, or tunica media of vasculature [15]. Clinically they appear as multiple skin coloured or pink-brown papules and nodules up to 2 cm in diameter, often around a hair follicle. Cold and pressure may trigger contraction and pain in 90% of cases.

HLRCC renal tumours have been shown to be significantly more aggressive than other renal cell carcinomas [4–10]. Renal tumours occur more commonly in women than men and present earlier than most renal cell carcinomas [5, 6]. Many patients present with metastatic disease and die less than 5 years from initial diagnosis. A recent study has also shown preliminary evidence of genetic anticipation, with reduced age onset of RCC in successive generations in four HLRCC families [12].

The lifetime risk of renal cancer is difficult to determine and varies widely between studies, ranging within 2%–20% [3, 6, 11–13, 16, 17]. This variation is most likely explained by the variety of recruitment methods used and the variable prevalence between different small cohorts.

Initial studies suggested that histologically HLRCC tumours were mostly type 2 papillary, although an expanding spectrum of histological architectural patterns have now been reported including papillary, tubulopapillary, tubular, solid, and cystic elements [5–7], as well as collecting-duct like carcinoma and sarcomatoid differentiation [6]. Most tumours are unilateral, solitary, and often asymptomatic although many patients present with metastatic disease. Symptoms include haematuria, low back pain, or a palpable mass. Early detection and surgical intervention of renal tumours are critically important. Prompt urology referral is advised in conjunction with a baseline abdominal CT/MRI and regular screening.

Management of HLRCC includes thorough assessment and screening for renal cell cancer, genetic counselling, and treatment of cutaneous lesions if they are multiple, disfiguring, or painful, alongside avoidance of painful triggers. Gynaecological input is also required to assess the severity of uterine leiomyomas. Additionally, malignant transformation to leiomyosarcomas may occur, although the risk of this is felt to be low [8]. There are no formal guidelines, but it is generally advised that patients undergo full skin, abdominal, and gynaecological examinations every 1-2 years. As the condition is inherited in an autosomal dominant pattern it is also vitally important to offer genetic counselling for family members.

Cutaneous lesions may be surgically excised but, as in our case, there is a high risk of recurrence, reported up to 15 years after excision. Electrodissection and cautery may be used for small lesions. Medical management includes neuropathic agents, topical analgesics, and carbon dioxide laser ablation [18]. Pain may be reduced by calcium channel blockers, doxazosin, phenoxybenzamine, and nitroglycerine which block smooth muscle contraction [19, 20]. Additionally botulinum toxin has also been shown to decrease the intensity of pain caused by leiomyomas [21].

## 5. Conclusions

The conclusions are as follows:

- HLRCC is an uncommon association between benign cutaneous leiomyomas, uterine leiomyomas, and renal cell cancer.
- Patients with HLRCC may present to the dermatologist with multiple tender, cutaneous lesions; diagnosis requires a high index of suspicion and careful elucidation of past and present medical problems.
- The most important aspect of diagnosis is thorough screening and monitoring for associated renal malignancy, which is extremely aggressive and a significant cause of mortality.
- All affected patients should be offered referral for genetic counselling.
